# Use of caliper-based external measurement of body surface in assessing the severity of pectus excavatum

**DOI:** 10.3389/fped.2022.1015026

**Published:** 2022-09-15

**Authors:** Tian Chen, Chenghao Chen, Qi Zeng, Yan Zhang, Jinghua Jiao, Xu Zhang, Na Zhang, Jie Yu

**Affiliations:** Department of Thoracic Surgery, Beijing Children's Hospital, Capital Medical University, National Center for Children's Health, Beijing, China

**Keywords:** pectus excavatum, external measurement, severity index, caliper, screening, follow-up

## Abstract

**Introduction:**

Objective assessment of the severity of pectus excavatum (PE) mainly depends on internal imaging examination, which poses radiation exposure risks and high financial costs. Our study explores the feasibility of caliper-based external measurements of the body surface to assess PE severity.

**Materials and methods:**

Patients with PE aged 4–18 years who underwent both internal imaging examinations and external measurements were chosen for the study. Overall, 176 patients underwent surgery and 21 underwent regular observation. The Haller index (HI) and correction index (CI) were used to derive the external measurement indices, HI-caliper and CI-caliper. Receiver-operator characteristic analysis provided the optimal cut-off values and compared the diagnostic values of HI-caliper and CI-caliper. Spearman's correlation coefficient and Cohen's kappa coefficient were used to analyze the correlation and consistency between HI-caliper or CI-caliper and HI-CT or CI-CT, respectively. Also, a paired samples *t*-test was used to compare the differences of HI-caliper or CI-caliper before and after surgery.

**Results:**

HI-caliper and CI-caliper measurements had strong correlations with HI-CT and CI-CT results (rs = 0.70, *p* < 0.001; rs = 0.69, *p* < 0.001), respectively. The optimal cut-off values of HI-caliper and CI-caliper were 1.83 (sensitivity = 0.841, specificity = 0.905) and 12% (sensitivity = 0.881, specificity = 0.857), exhibiting comparable diagnostic values with HI-CT and CI-CT. HI-caliper > 1.83 or CI-caliper > 12% had medium intensity consistency with HI-CT ≥ 3.25 or CI-CT ≥ 28% (*k* = 0.545, 95% confidence interval: 0.374–0.716, *p* < 0.001). The HI-caliper and CI-caliper values were significantly different before and after surgery.

**Conclusion:**

Caliper-based external measurement is a feasible method to screen patients who require surgical intervention and for monitoring the progression of PE severity.

## Introduction

Pectus excavatum (PE) is the most common congenital chest wall deformity caused by costal cartilage overgrowth. It manifests as a depression of the anterior chest wall, which may adversely affect physical appearance, cardiopulmonary function, and mental health (1). Currently, the use of different therapeutic interventions is determined by the severity of PE, and the objective assessment of PE severity mainly depends on the internal measurement indices: the Haller index (HI) and the correction index (CI) (2, 3). Therefore, internal imaging examinations are indispensable in the diagnosis and follow-up of PE. Routine examination of patients with PE by computed tomography (CT) can completely present the chest wall structure and accurately assess the severity of the condition, guiding treatments such as functional exercise, vacuum bells, and surgical intervention (4). However, when assessing the severity of PE, the HI and CI indices are only measured at the cross-section of the deepest sternal depression observed by multiple CT scan slices. Hence, the value of the measurements obtained from CT is not in proportion to the radiation risk and financial burden imposed by the method. Considering that body surface morphology can reflect the thoracic bone structure to a certain extent, we propose a caliper-based external measurement method to assess the severity of PE. This study attempts to validate the feasibility and accuracy of caliper-based external measurement indices in assessing the severity of PE by comparing them with CT-based internal measurement indices. We aim to demonstrate the external measurement of the body surface as an alternative to the internal measurement of the bony thorax for preliminary screening of patients with PE who need surgical intervention and subsequent follow-up.

## Materials and methods

### Study design

This study was approved by the institutional review board ([2022]-E-113-R) and informed consent was obtained from the parents of each patient. Patients with PE aged 4–18 years who visited Beijing Children's Hospital from May 2021 to September 2021 were included in this cross-sectional study. The study population consisted of two groups: the first group included inpatients with moderate-to-severe PE who underwent a minimally invasive surgical repair called the Nuss procedure (operation group). Meanwhile, the second group included outpatients with mild PE who underwent functional exercise or a vacuum bell, and they were regularly observed and followed up every 6 months until the depression was severe enough to be treated surgically (non-operation group).

### Severity indices

**Figure 1:**
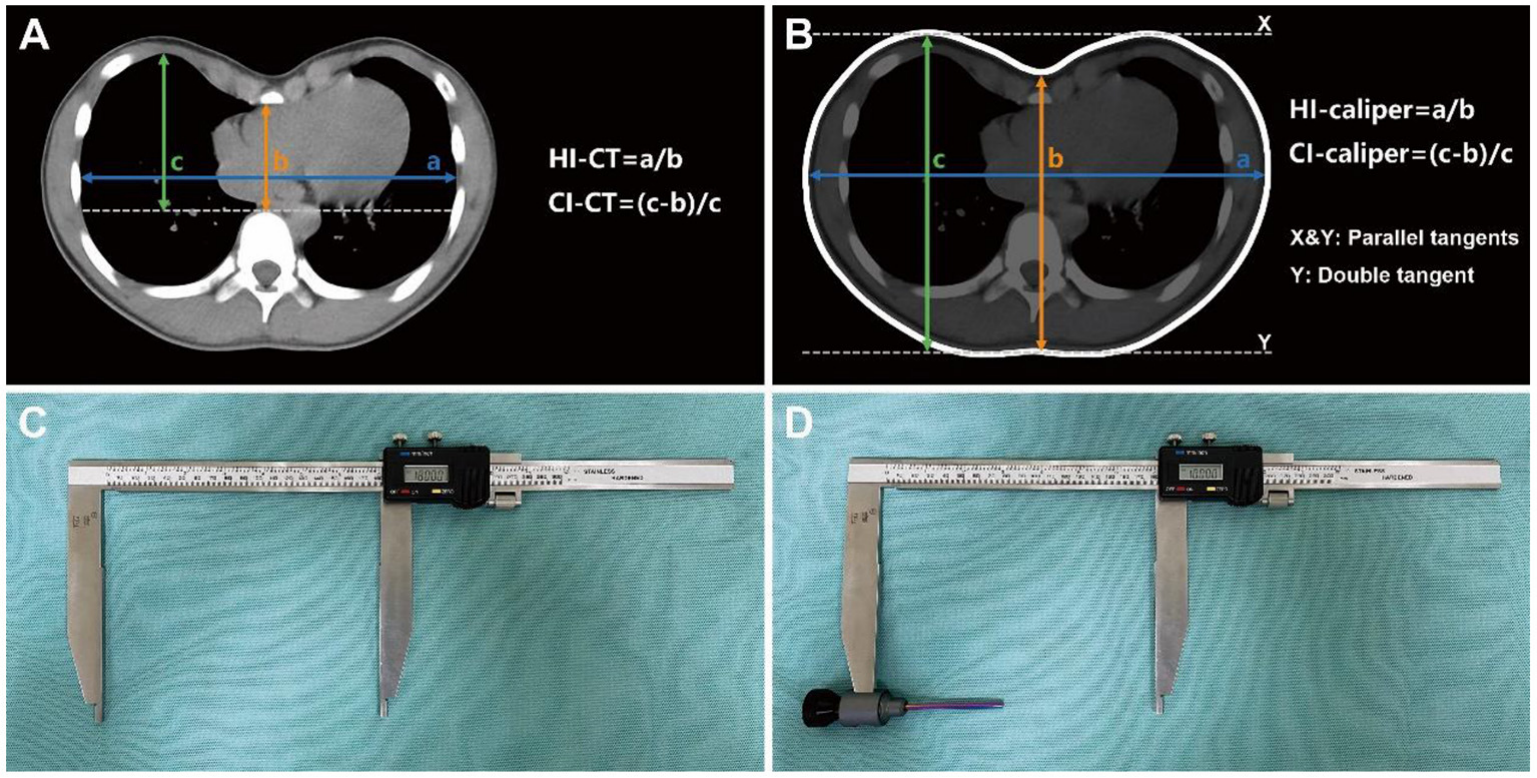
Severity indices and measuring caliper. **(A)** CT-based severity indices of internal measurement, targeting the bony thorax from pleura to pleura. **(B)** Caliper-based severity indices of external measurement, targeting the body surface from skin to skin. **(C)** Caliper for measuring the maximum distance between the parallel tangents of bilateral chest wall (a in **B**) and the maximum distance between the parallel tangents of anterior-posterior chest wall (c in **B**). **(D)** Caliper with “probe” installed for measuring the minimum distance from the deepest point of anterior chest wall depression to the double tangent of bilateral posterior chest wall (b in **B**).

At the cross-section of the deepest sternal depression, the CT-based internal measurement index HI was defined as the ratio of the maximum internal transverse diameter to the minimum anterior-posterior diameter from the back of the sternum to the front of the vertebral body. Surgical intervention was indicated for patients with HI ≥ 3.25 (Figure 1A) (2). The CI is defined as the ratio of the depression depth to the maximum distance between the inner edge of the anterior chest wall and the horizontal tangent of the front of the vertebral body, whereas the depression depth is the difference between the maximum distance from the inner edge of the anterior chest wall to the horizontal tangent and the minimum distance from the back of the sternum to the horizontal tangent. Surgical intervention was indicated for patients with CI ≥ 28% (Figure 1A) (3, 5).

The caliper-based external measurement indices, HI-caliper and CI-caliper, were derived and defined according to the CT-based internal measurement indices, HI-CT and CI-CT, respectively. The horizontal plane of the deepest depression on the body surface was selected for measurement, whereas the distances used for calculation were from skin to skin. The HI-caliper was defined as the ratio of the maximum distance between the parallel tangents of the bilateral chest wall to the minimum distance from the deepest point of the anterior chest wall depression to the double tangent of the bilateral posterior chest wall (Figure 1B). The CI-caliper was defined as the ratio of the depression depth to the maximum distance between the parallel tangents of the anterior-posterior chest wall, whereas the depression depth was defined as the maximum distance between the parallel tangents of the anterior-posterior chest wall minus the minimum distance from the deepest point of the anterior chest wall depression to the double tangent of the bilateral posterior chest wall (Figure 1B). Parallel tangents were two parallel lines, respectively, tangent to the bilateral sides or to the anterior-posterior sides of the chest wall. Double tangent was a line simultaneously tangent to the bilateral sides of the back (Figure 1B).

### Internal measurement

For patients with moderate-to-severe PE who underwent surgery, CT was employed as a routine examination to assess the PE severity, evaluate the surgical risk, and guide the surgical plan. Meanwhile, for patients with mild PE who underwent regular observation and follow-up, CT was employed as a screening method to assess the severity and decide whether surgery was required. However, some patients with mild PE were judged by physical examination to have an anterior chest wall depression that was too shallow; hence, no further CT was performed. To avoid image artifacts during the CT examination, patients were required to remain in a supine position with their arms raised and maintain quiet breathing. The CT images of patients were acquired from the Picture Archiving and Communication System. The HI and CI were measured and calculated by a single thoracic surgeon, who was blinded to the study, using standard methods with the help of an electronic caliper in the imaging system.

### External measurement

**Figure 2:**
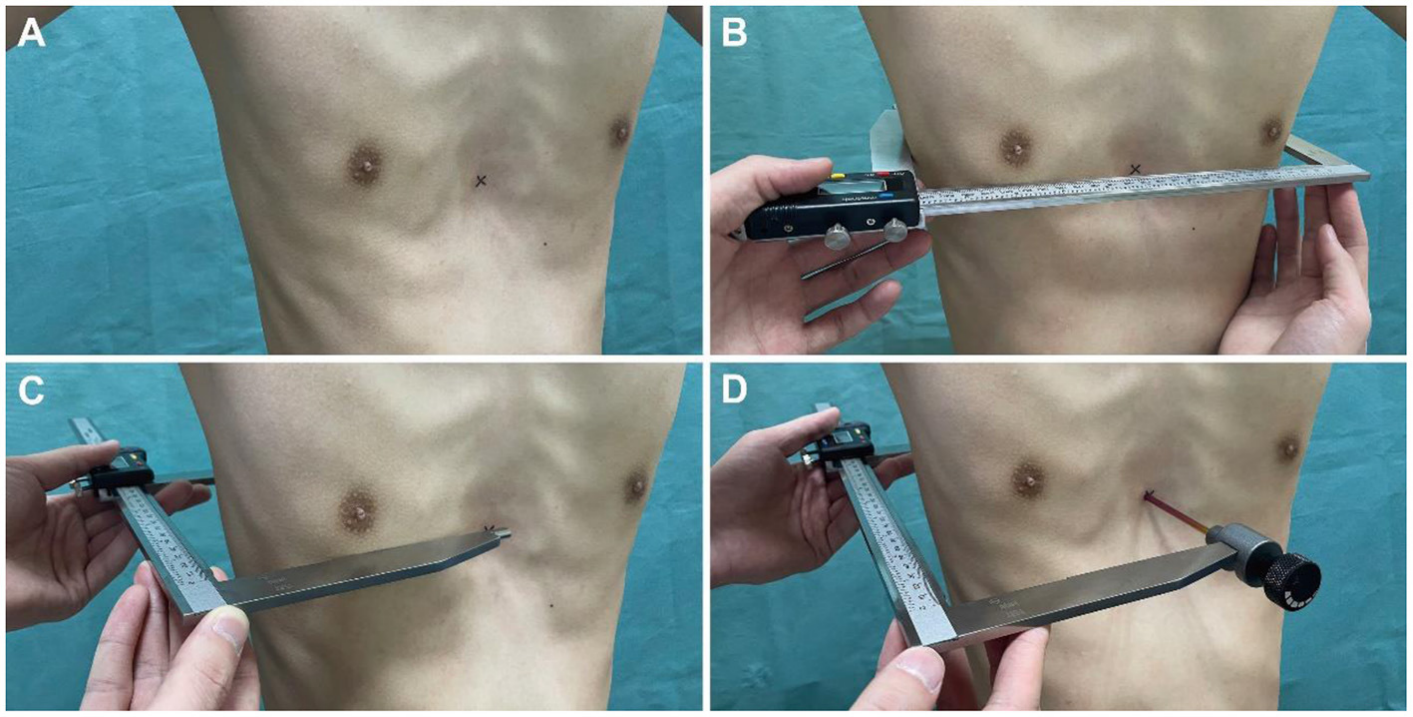
Methods of caliper-based external measurement. **(A)** Marked the deepest point of anterior chest wall depression on the body surface with an erasable marker. **(B)** On horizontal plane of the deepest depression of anterior chest wall, measured the maximum distance between the parallel tangents of bilateral chest wall (a in Figure 1B). **(C)** Measured the maximum distance between the parallel tangents of anterior-posterior chest wall (c in Figure 1B). **(D)** Measured the minimum distance from the deepest point of anterior chest wall depression to the double tangent of bilateral posterior chest wall (b in Figure 1B).

Caliper-based external measurements were employed as routine physical examinations for all patients. Inpatients in the operation group were assessed on the day before surgery and on the fourth day after surgery. Outpatients in the non-operation group were assessed immediately at their visit. Also, external measurements on horizontal plane of the lower sternum in a subset of individuals presenting for non-chest wall deformities were consecutively collected. To avoid post-operative wound pain or dehiscence during external measurement, patients were asked to remain in a standing position with their arms lifted at 45° and maintain quiet breathing. The caliper used for the external measurement was a commercial Vernier caliper (Hengliang, 111-501, Shanghai, China) with an accuracy of 0.01 mm and an error of ±0.05 mm (Figure 1C). Since the Vernier caliper could only measure the distance between the convex surfaces of the body, a detachable “probe” was installed on the upper measuring claw to detect the depression. The “probe” did not affect the measurement accuracy as the electronic Vernier caliper could be reset to zero and complete the measurement whether the “probe” was installed or not (Figure 1D). The HI-caliper and CI-caliper were measured and calculated by another single thoracic surgeon, who was blinded to the study, through the defined methods utilizing the caliper (Figure 2).

### Statistical analysis

Descriptive statistics were calculated for demographics along with caliper-based and CT-based measurements. Spearman's correlation coefficient was used to analyze the correlation between the HI-caliper and HI-CT, as well as the CI-caliper and CI-CT. Receiver-operator characteristic (ROC) analysis was used to obtain the optimal cut-off values and analyze the sensitivity and specificity of HI-caliper and CI-caliper values for distinguishing patients who had PE and for screening patients who needed surgical intervention. Pairwise comparisons of ROC curves were used to compare the diagnostic values of HI-caliper, CI-caliper, HI-CT, and CI-CT. Under the respective optimal cut-off value, Cohen's kappa coefficient was used to analyze the consistency of HI-caliper or CI-caliper and HI-CT or CI-CT for screening patients. A paired samples *t*-test was used to reflect the significance of HI-caliper and CI-caliper in quantifying the improvement in severity before and after surgery. Statistical analyses were performed using SPSS 19.0 (IBM, Armonk, New York) and MedCalc 19.0.4 (MedCalc Software, Ostend, Belgium).

## Results

Overall, 182 patients were consecutively included in the operation group, of whom 6 were excluded due to the lack of CT images in our hospital. Meanwhile, 34 patients were consecutively included in the non-operation group, of whom 13 were excluded because the anterior chest wall depression was too shallow, as determined by physical examination, and no further CT was performed. Finally, in the operation group of 176 patients, the pre-operative median HI-caliper was 1.99 (range: 1.37–2.94) vs. the post-operative value of 1.65 (range: 0.90–2.03). The pre-operative median CI-caliper was 17.43% (range: 6.12–45.12%) vs. the post-operative value of 4.49% (range: −0.96–11.27%). In the non-operation group of 21 patients, the median HI-caliper was 1.73 (range: 1.42–1.88) and the median CI-caliper was 9.11% (range: 2.97–17.33%) (Table 1). Additionally, of 34 patients with mild PE, the median HI-caliper was 1.75 (range: 1.42–2.00) and the median CI-caliper was 9.05% (range: 2.97–17.33%); and of 36 individuals with non-PE, the median HI-caliper was 1.41 (range: 1.25–1.77) and the median CI-caliper was 2.85% (range: 2.59–6.97%) (Table 2).

**Table 1:** Demographics and measurements stratified by study groups.

**Variable**	**Operation group**	**Non-operation group**
Patient	176	21
Age (years, months)	12.10 (4.2–17.5)	8.4 (6.0–15.11)
Sex (male)	138 (78.4%)	14 (66.7%)
Body mass index	15.7 (11.1–24.1)	15.4 (11.1–26.8)
**Haller index**
Pre-op CT	3.88 (2.34–20.46)	2.94 (2.25–3.85)
Pre-op caliper	1.99 (1.37–2.94)	1.73 (1.42–1.88)
Post-op caliper	1.65 (0.90–2.03)	—
**Correction index (%)**
Pre-op CT	30.83 (10.69–82.72)	17.33 (8.44–30.93)
Pre-op caliper	17.43 (6.12–45.12)	9.11 (2.97–17.33)
Post-op caliper	4.49 (−0.96–11.27)	—

Continuous variables listed as median (range), categorical variables listed as n (%).

**Table 2:** Demographics and measurements stratified by population groups.

**Variable**	**Mild PE patients**	**Non-PE individuals**
Patient	34	36
Age (years, months)	8.8 (4.0–15.11)	7.5 (4.0–15.6)
Sex (male)	21 (61.8%)	19 (52.8%)
Body mass index	15.5 (11.1–27.3)	16.6 (10.0–23.0)
HI-caliper	1.75 (1.42–2.00)	1.41 (1.25–1.77)
CI-caliper (%)	9.05 (2.97–17.33)	2.85 (2.59–6.97)

Continuous variables listed as median (range), categorical variables listed as n (%).

**Figure 3:**
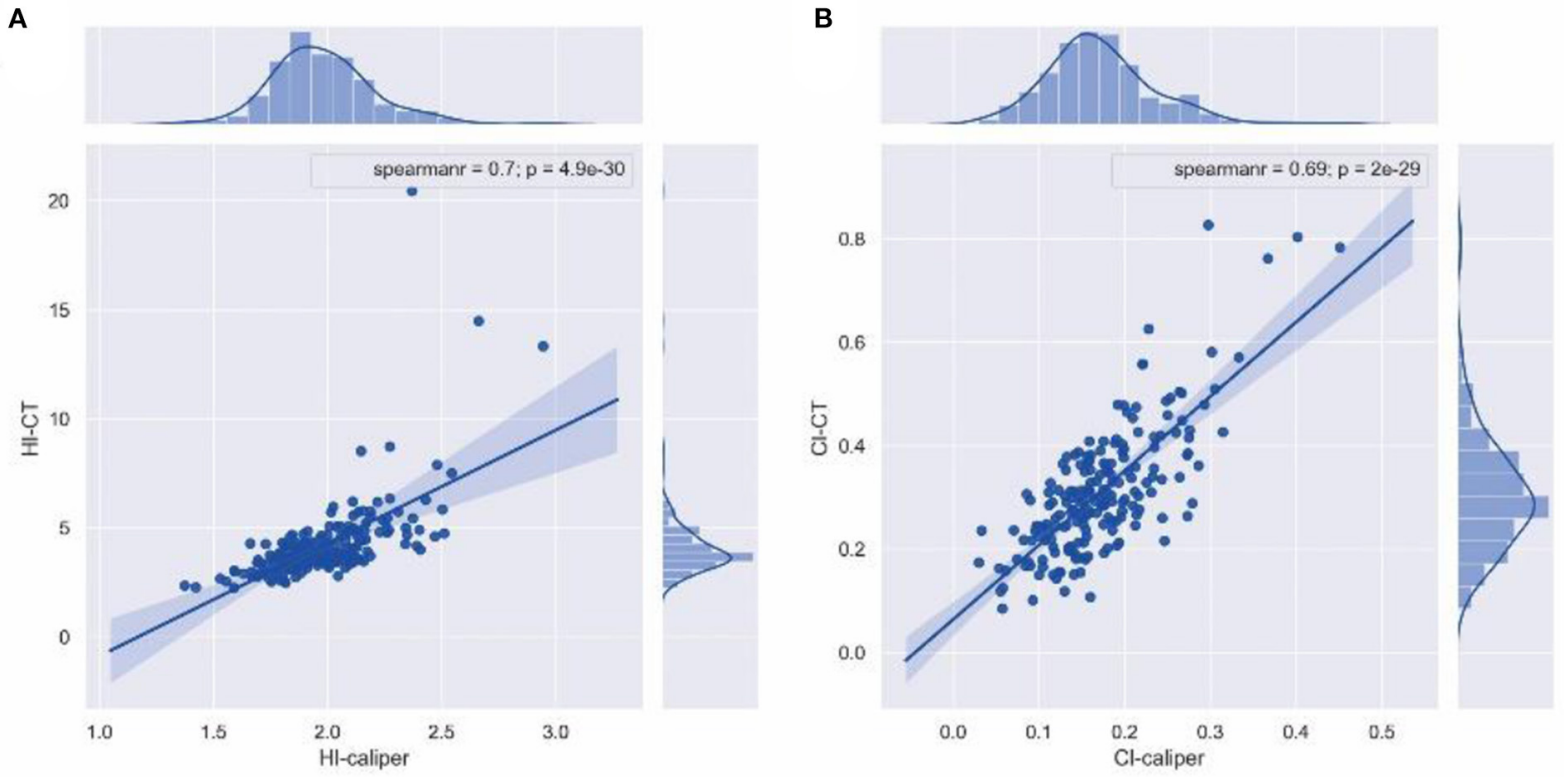
Spearman's correlation coefficient. **(A)** HI-caliper was strongly correlated with HI-CT (rs = 0.70, *p* < 0.001). **(B)** CI-caliper was strongly correlated with CI-CT (rs = 0.69, *p* < 0.001).

Spearman's correlation coefficient of 176 patients in the operation group and 21 patients in the non-operation group revealed that HI-caliper was strongly correlated with HI-CT (rs = 0.70, *p* < 0.001), while CI-caliper was strongly correlated with CI-CT (rs = 0.69, *p* < 0.001) (Figure 3).

**Figure 4:**
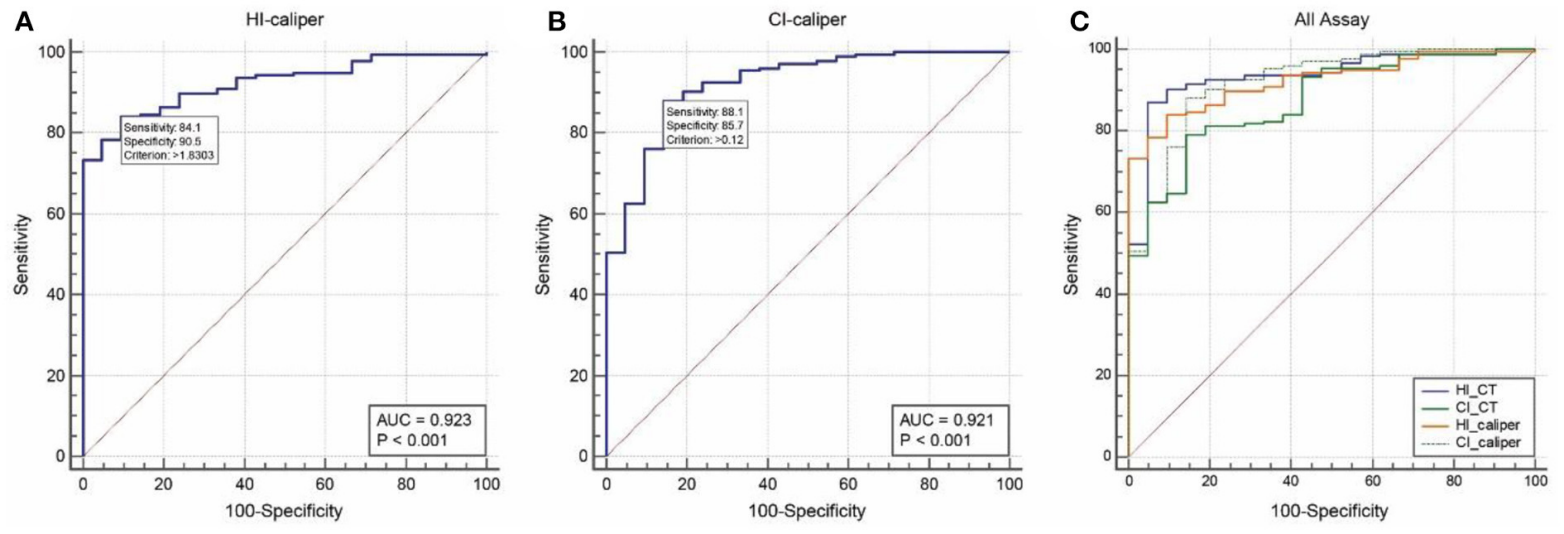
Receiver-operator characteristic analysis. **(A)** The area under the curve (AUC) and optimal cut-off value of the HI-caliper were 0.923 (95%CI: 0.877–0.956, *p* < 0.001) and 1.83 (sensitivity = 0.841, specificity = 0.905), respectively. **(B)** The AUC and optimal cut-off value of the CI-caliper were 0.921 (95%CI: 0.874–0.955, *p* < 0.001) and 12% (sensitivity = 0.881, specificity = 0.857), respectively. **(C)** The AUC of HI-CT was 0.935 (95%CI: 0.891–0.965, *p* < 0.001), with a sensitivity of 0.894 and a specificity of 0.905 under HI-CT = 3.25. The AUC of CI-CT was 0.874 (95%CI: 0.820–0.917, *p* < 0.001), with a sensitivity of 0.625 and a specificity of 0.912 under CI-CT = 28%.

ROC analysis of 176 patients in the operation group and 21 patients in the non-operation group showed that the area under the curve (AUC) and optimal cut-off value of the HI-caliper were 0.923 (95%CI: 0.877–0.956, *p* < 0.001) and 1.83 (sensitivity = 0.841, specificity = 0.905), respectively. The AUC and optimal cut-off value of the CI-caliper were 0.921 (95%CI: 0.874–0.955, *p* < 0.001) and 12% (sensitivity = 0.881, specificity = 0.857), respectively. Meanwhile, the AUC of HI-CT was 0.935 (95%CI: 0.891–0.965, *p* < 0.001) with a sensitivity of 0.894 and a specificity of 0.905 under HI-CT = 3.25. The AUC of CI-CT was 0.874 (95%CI: 0.820–0.917, *p* < 0.001) with a sensitivity of 0.625 and a specificity of 0.912 under CI-CT = 28% (Figure 4). According to the pairwise comparison of ROC curves, HI-caliper or CI-caliper had no statistical difference with either HI-CT or CI-CT in diagnostic value.

Setting HI-caliper > 1.83 or CI-caliper > 12% as the external measurement criteria for screening patients with PE who needed surgical intervention, Cohen's kappa coefficient revealed that these criteria had medium intensity consistency with the radiological criteria of HI-CT ≥ 3.25 or CI-CT ≥ 28% (*k* = 0.545, 95%CI: 0.374–0.716, *p* < 0.001). Significantly, considering whether patients underwent surgical intervention as the gold standard, the sensitivity, specificity, and accuracy of the external measurement criteria of HI-caliper > 1.83 or CI-caliper > 12% were 0.972, 0.762, and 0.949, respectively.

ROC analysis of 34 patients with mild PE and 36 individuals with non-PE showed that the AUC and optimal cut-off value of the HI-caliper were 0.945 (95%CI: 0.863–0.985, *p* < 0.001) and 1.51 (sensitivity = 0.941, specificity = 0.917), respectively. The AUC and optimal cut-off value of the CI-caliper were 0.953 (95%CI: 0.875–0.989, *p* < 0.001) and 5% (sensitivity = 0.912, specificity = 0.944), respectively.

**Figure 5:**
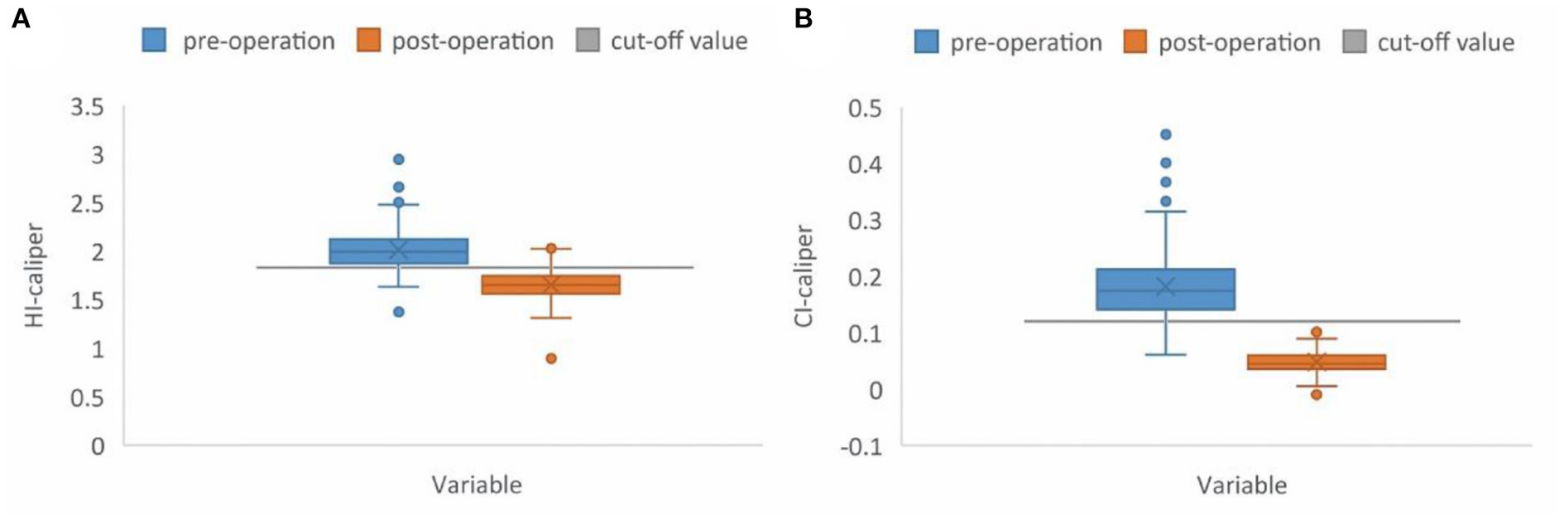
Box plot. **(A)** The preoperative median HI-caliper was 1.99 (range: 1.37–2.94), vs. the post-operative value of 1.65 (range: 0.90–2.03). The post-operative HI-caliper, of 154 patients (87.5%), recovered and dropped below the cut-off value of 1.83. **(B)** The preoperative median CI-caliper was 17.43% (range: 6.12–45.12%), vs. the post-operative value of 4.49% (range: −0.96–11.27%). The post-operative CI-caliper of all patients (100.0%) recovered and dropped below the cut-off value of 12%.

A paired samples *t*-test, of 176 patients in the operation group, showed a significant difference between the pre-operative and post-operative HI-caliper (*t* = 25.00, *p* < 0.001). The post-operative HI-caliper of 154 patients (87.5%) recovered and dropped below the cut-off value of 1.83. Meanwhile, a significant difference between the pre-operative and post-operative CI-caliper was observed (*t* = 30.08, *p* < 0.001), and the post-operative CI-caliper of all patients (100.0%) recovered and dropped below the cut-off value of 12% (Figure 5).

## Discussion

Currently, children with PE need different therapeutic interventions according to their severity; however, there has been extensive and continuous discussions on how to objectively assess the severity of PE. CT-based HI and CI are the most widely accepted and used quantitative indices of severity and are regarded as the main surgical indications of minimally invasive surgical repair for patients with PE (6). However, CT should not be used repeatedly for the same child due to the risk of radiation exposure. Therefore, an accurate, repeatable, safe, and affordable assessment method to preliminarily screen children with PE to determine the need for surgical intervention and for subsequent follow-up to quantify the exacerbation or improvement of PE would benefit a broad population of children with PE (7).

To quantify and assess the severity of PE, surgeons initially recorded the chest wall morphology by simply measuring the thoracic diameters using measuring rulers. In 1978, Haller developed a spatial planimetry to record the body surface arcs of different horizontal planes of the chest wall, achieving a spatial visualization (8). In 1981, Hecker simplified the concept to reduce the financial cost of measurement. Several key points of the chest wall were measured using a pelvic measuring caliper, and these key points were connected to draw and record the body surface arcs (9). Later, with the development of imaging examination, the assessment of PE severity changed from the external measurement of the body surface (skin to skin) to the internal measurement of the bony thorax (pleura to pleura). In 1987, Haller proposed the CT-based severity index of HI, which is widely used today (2).

Considering the radiation risk and financial cost, surgeons question the reasonability of CT examination and have attempted to find an alternative method for assessing PE severity. The feasibility of using X-rays, magnetic resonance (MR), and optical scanners have been explored to assess the severity of PE (10–12). MR and optical scanners do not involve the risk of radiation exposure; however, MR involves high financial cost, long examination time, and contraindication regarding metal implants in the body, which is hardly applicable for children with PE (11, 13). Optical scanners require complex equipment and specialized technicians, which cannot identify the internal conditions of the thorax, and the cost of repeated monitoring during the progression of PE can be overwhelming (7, 14).

When assessing the severity of PE, whether by CT, MR, or optical scanner, the severity indices are measured and calculated only on the cross-section of the deepest depression. Meanwhile, the internal diameters of the bony thorax are correlated with the corresponding external diameters of the body surface. Therefore, some surgeons have simplified the measurement concepts and proposed to externally measure the body surface using a caliper to quantify and assess the severity of PE (7, 15–17).

In our study, we utilized a modified Vernier caliper for external measurement. The reproducibility of measurement depends on the accuracy and error of the caliper, not the “probe”, and any similar patented or commercial Vernier caliper could be utilized. When the two parallel measuring claws of the caliper are tangent to the arcs of the body surface, the distance between the measuring claws represents the corresponding thoracic diameter. By using the Vernier caliper and measuring along the thoracic horizontal plane, the maximum and minimum diameters used for calculating the HI-caliper and CI-caliper can be measured objectively, which avoids visual deviation.

We demonstrated that the external measurement indices, HI-caliper and CI-caliper, strongly correlated with the internal measurement indices HI-CT and CI-CT, respectively. Additionally, we found the optimal cut-off values of HI-caliper and CI-caliper to distinguish whether surgical intervention is needed and verified that their diagnostic values were not significantly different from HI-CT and CI-CT. Considering that the calculation of HI-caliper includes the transverse diameter of the thorax and is more suitable for patients with flat chests and CI-caliper reflects the depth of chest wall depression and is more suitable for PE that manifests as depression, we recommend setting HI-caliper > 1.83 or CI-caliper > 12% as the criteria for preliminary screening of patients with PE who need surgical intervention. These criteria had a satisfactory accuracy and a medium intensity consistency with HI ≥ 3.25 or CI ≥ 28%. Also, HI-caliper > 1.51 or CI-caliper > 5% could be the criteria to distinguish patients who had flat or depressed deformities. Additionally, we validated that HI-caliper or CI-caliper could significantly reflect the difference in severity before and after surgery.

During the study period, comorbidities identified when patients (*n* = 197) underwent CT examination due to chest wall depression included: emphysema (*n* = 4), bifid rib (*n* = 2), congenital cystic lung malformation (*n* = 1), pulmonary bulla (*n* = 1), bronchial diverticulum (*n* = 1), subpleural nodule (*n* = 1), dextrocardia (*n* = 1), pericardial effusion (*n* = 1), and patent ductus arteriosus (*n* = 1). Thus, pre-operative CT examination can distinguish the comorbidities of PE, such as congenital cystic lung malformation, and the associated surgical risk factors, such as dextrocardia. Additionally, some surgeons reconstruct the chest wall structure and conduct mechanical analysis to optimize the implantation plan of the Nuss bar through CT images. Therefore, a single pre-operative CT examination should be typically ordered for pre-operative assessment in patients with moderate to severe PE.

However, the younger the patient is when undergoing CT, the greater the risk of cancer in the future (18, 19). Radiation doses from CT scans ought to be kept as low as possible and alternative procedures, which do not involve ionizing radiation, should be considered if appropriate (20). The monitoring and treatment of PE is a long-term process that lasts for several years, and the severity may need to be assessed several times in this process. Due to the lack of an ideal method, we were forced to obtain HI and CI through CT to assess the severity. Of the 34 patients ultimately determined as mild PE in this study, 13 patients with particularly shallow depression were judged not to need surgery through physical examination. However, the other 21 patients had to obtain HI and CI through CT examination to assist surgeons in determining whether the depression was serious enough to meet the surgical indications, and they were potential candidates for multiple CT scans. Therefore, approximately two-thirds of patients with mild PE could have avoided unnecessary radiation by replacing CT-based measurements with caliper-based measurements.

As an objective evaluation method of PE severity, caliper-based external measurement can be repeatedly utilized in monitoring the progression of pre-operative severity, preliminarily screening patients who need surgical intervention, and quantifying the improvement in post-operative severity. Moreover, for patients with mild PE treated with functional exercise or a vacuum bell, caliper-based external measurement provides an excellent monitoring method for documenting the severity of depression that changes with treatment, which is acceptable and affordable for patients. Accordingly, in future preliminary screening, when patients meet the external measurement criteria of HI-caliper > 1.83 or CI-caliper > 12%, which indicates sufficient severity and need of surgery, further CT examination can be performed to distinguish the comorbidities and surgical risks to guide the surgery. Conversely, patients who do not meet these criteria should be closely observed and regularly monitored for severity by caliper-based external measurement.

Although we have confirmed that the caliper-based external measurement is an effective assessment method, there are still limitations in the study. First, the reproducibility between multiple measurements of the same patient by different surgeons had not been established. Significantly, our finding of an optimal cut-off value of CI-caliper > 12% is consistent with the findings in an approximate study by Rebeis, which may indirectly reflect the reproducibility between different testers (15). Second, the assessment method might be influenced by potential distortion of soft tissue, such as female breast development and patient body mass index. The indices were the ratios of thoracic diameters, which might partially eliminate the influence of soft tissue. Nevertheless, further studies stratified by sex and body mass index are needed. Finally, the conclusions of the study need to be supported by a larger sample size and validated in a broad population of patients with PE. However, we believe that caliper-based external measurements are an effective method for the assessment of PE severity, and can be generalized and profoundly beneficial for patients with PE.

## Conclusion

Transitioning from CT-based internal measurements to caliper-based external measurements provides a radiation-free method to assess the severity of PE during preliminary screening and subsequent follow-up. The method is feasibility and accuracy, and setting HI-caliper > 1.83 or CI-caliper > 12% as the criteria for screening patients with PE who need surgical intervention is recommended. However, its widespread application in clinical procedure still requires support and verification in a broad population.

## Data availability statement

The raw data supporting the conclusions of this article will be made available by the authors, without undue reservation.

## Ethics statement

Written informed consent was obtained from the minor(s)' legal guardian/next of kin for the publication of any potentially identifiable images or data included in this article.

## Author contributions

TC conceived and designed the study, analyzed and interpreted the data, and drafted the manuscript. CC analyzed and interpreted the data and revised the manuscript critically for intellectual content. QZ performed the final approval of the version to be submitted. YZ, JJ, and XZ acquired the data. NZ and JY conceived and designed the study. All authors have read and approved the final manuscript.

## Conflict of interest

The authors declare that the research was conducted in the absence of any commercial or financial relationships that could be construed as a potential conflict of interest.

## Publisher's note

All claims expressed in this article are solely those of the authors and do not necessarily represent those of their affiliated organizations, or those of the publisher, the editors and the reviewers. Any product that may be evaluated in this article, or claim that may be made by its manufacturer, is not guaranteed or endorsed by the publisher.

## Abbreviations

AUC: Area under the curve
CI: Correction index
CT: Computed tomography
HI: Haller index
MPD: Modified percent depth
MR: Magnetic resonance
PE: Pectus excavatum
ROC: Receiver-operator characteristic.
